# Micronization of Taxifolin by Supercritical Antisolvent Process and Evaluation of Radical Scavenging Activity

**DOI:** 10.3390/ijms13078869

**Published:** 2012-07-16

**Authors:** Shuchong Zu, Lei Yang, Jinming Huang, Chunhui Ma, Wenjie Wang, Chunjian Zhao, Yuangang Zu

**Affiliations:** State Engineering Laboratory for Bio-Resource Eco-Utilization, Northeast Forestry University, Harbin, 150040, China; E-Mails: zushuchong@163.com (S.Z.); ylhjmnefu@163.com (J.H.); mchmchmchmch@163.com (C.M.); wjwang225@hotmail.com (W.W.); zcjsj@163.com (C.Z.)

**Keywords:** taxifolin, micronization, supercritical antisolvent, dissolution rate, radical scavenging activity, *Larix gmelinii*

## Abstract

The aim of this study was to prepare micronized taxifolin powder using the supercritical antisolvent precipitation process to improve the dissolution rate of taxifolin. Ethanol was used as solvent and carbon dioxide was used as an antisolvent. The effects of process parameters, such as temperature (35–65 °C), pressure (10–25 MPa), solution flow rate (3–6 mL/min) and concentration of the liquid solution (5–20 mg/mL) on the precipitate crystals were investigated. With a lower temperature, a stronger pressure and a lower concentration of the liquid solution, the size of crystals decreased. The precipitation temperature, pressure and concentration of taxifolin solution had a significant effect. However, the solution flow rate had a negligible effect. It was concluded that the physicochemical properties and dissolution rate of crystalline taxifolin could be improved by physical modification such as particle size reduction using the supercritical antisolvent (SAS) process. Further, the SAS process was a powerful methodology for improving the physicochemical properties and radical scavenging activity of taxifolin.

## 1. Introduction

Taxifolin, 3,3′,4′,5,7-pentahydroxiflavanon, is an active compound isolated from the xylem of *Larix gmelinii* [1,2]. It’s also found in the açaí palm, in milk thistle seeds and in small quantities in red onion. Taxifolin has a positive effect on human health, as it prevents accumulation of free radicals [3,4], influences the physical properties of lipids in biological membranes [5], ameliorates cerebral ischemia-reperfusion injury [6] and activates the formation of collagen fibers [7]. Application of taxifolin is quite widely distributed in the production of different categories of products. In general, taxifolin can be used as a natural antioxidant additive in the food industry [1].

The poor solubility of active pharmaceutical ingredients in water and their low dissolution rate often leads to insufficient bioavailability, which is one of the most difficult and non-solved problems in pharmaceutical technology. Taxifolin is a natural compound with poor solubility, which leads to a low bioavailability [8]. This drawback limits the medical development as well as the use of food, health products and cosmetics.

Reducing drug particle size is an effective and widely used approach to increase the solubility by enlarging the effective surface area. Besides the conventional methods, *i.e.*, spray-drying and solvent evaporation, new technologies have recently been developed to improve wettability and aqueous solubility of active pharmaceutical ingredient. These methods apply new concepts based on the use of compressed gases, supercritical fluids or liquefied gases as solvents [9].

Supercritical fluid technology has been applied for micronization, extraction, coating, separation, chemical reaction, *etc.* [10–13]. In recent years, particle formation using supercritical fluid has been extensively discussed because of its operational flexibility and environmental compatibility in such processes [14]. Although various particle formation techniques using supercritical fluid can be chosen [15], the supercritical antisolvent (SAS) process is the one most widely used in the literature to produce microparticles of various organic and inorganic compounds [11,16–18]. Many researchers have employed the SAS process for micronization and recrystallization of various pharmaceutical substances.

The major advantages of the SAS process, such as easy handling of difficult-to-comminute materials, use of a nontoxic medium, and a mild operating temperature, may provide ideal conditions for the processing of pharmaceutical compounds [19]. Carbon dioxide is a widely used supercritical fluid because of its relatively low critical temperature, pressure and nontoxic property [20,21]. Moreover, CO_2_ is gaseous at ambient conditions, which simplifies the problem of solvent residues [22].

Our research is aimed to improve the antioxidant activities of taxifolin by increasing its solubility using SAS technology. The parameters of the SAS process of how it affects the morphology and particle size are studied. Moreover, unprocessed and processed taxifolin is characterized by scanning electron microscopy (SEM), thermal gravimetric analysis (TG), Fourier-transform infrared spectroscopy (FTIR), and specific surface area (BET) analyses. The dissolving capability and 2,2-diphenyl-1-picrylhydrazyl (DPPH) radical-scavenging activity are also evaluated.

## 2. Results and Discussion

### 2.1. Morphology

**Figure 1:**
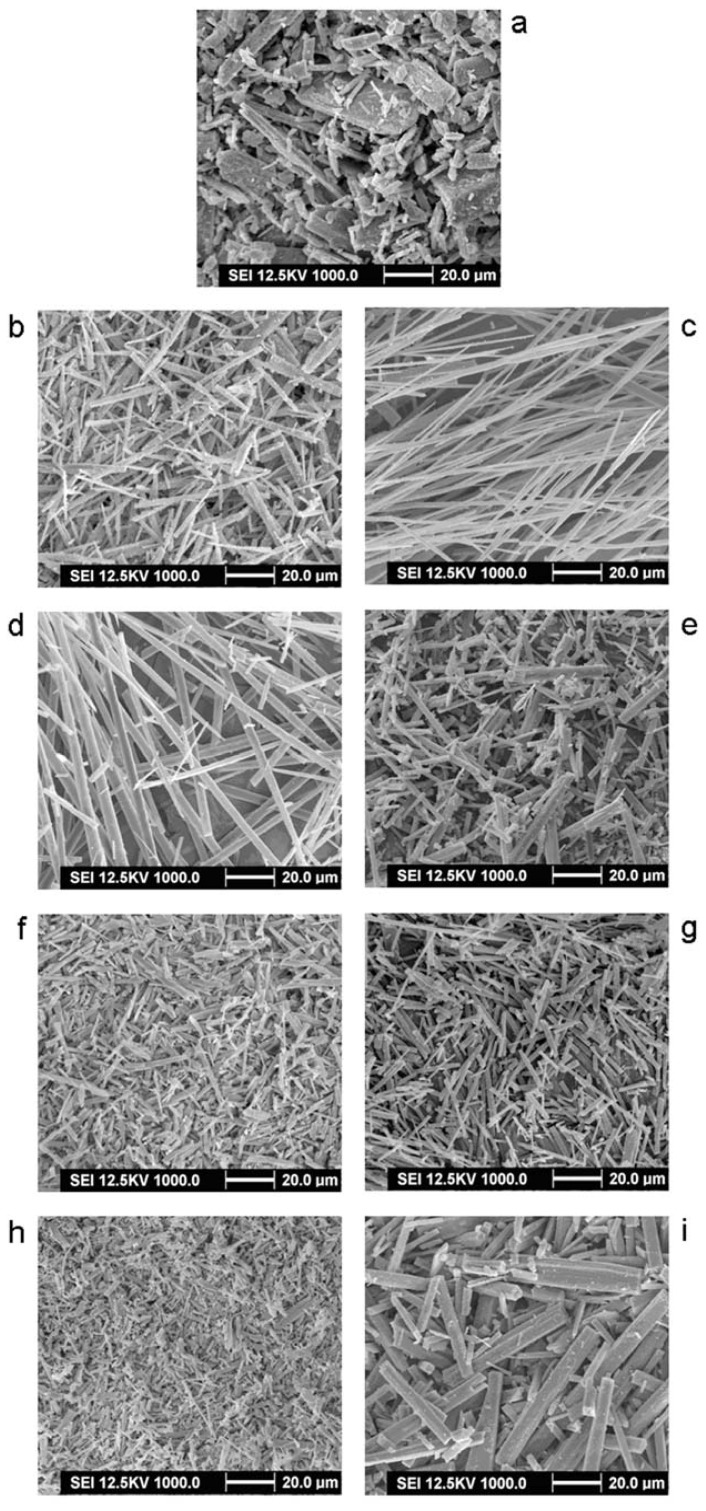
(**a**) Supercritical antisolvent (SEM) image of unprocessed taxifolin; (**b**), (**c**), (**d**), (**e**), (**f**), (**g**), (**h**) and (**i**) SEM images of processed taxifolin from Trial no.1, 4, 5, 7, 8, 10, 11 and 13 (in Table 1).

The comparison between unprocessed and processed taxifolin particles can be seen in Figure 1. The SEM micrographs of unprocessed taxifolin showed an irregularly shape (Figure 1a). The effects of temperature, pressure, concentration and solution flow rate on the characteristics of the taxifolin were examined.

The effect of temperature is a vital process parameter for the size of fibers in the SAS process. The other parameters were carried out at a pressure of 20 MPa, a taxifolin concentration 10 mg/mL and a solution flow rate of 4 mL/min. With increasing the temperature from 35 to 65 °C, taxifolin was precipitated in the form of needle-like fibers ranging from 5–30 μm and 40–200 μm in length. As shown in Figure 1b,c, the size of processed taxifolin increased as temperature increased. The morphology change of the taxifolin was can probably be explained in terms of volume expansion [23,24]. In general, at a low temperature, volume expansion is faster than at high temperature, so smaller fibers were obtained at 35 °C in the SAS process.

We discuss a series of SAS experiments at different pressures acting on the powder morphology and size. The other operation parameters were set at: temperature of 35 °C, solution concentration of 10 mg/mL and solution flow rate of 4 mL/min. As shown in Figure 1d,e, the size of processed taxifolin was reduced from 10–300 μm at 10 MPa to 2–10 μm at 25 MPa. When the pressure was 10 MPa, long needle shaped crystals of taxifolin several microns in length were obtained (Figure 1d). As the pressure increased to 25 MPa, narrower and shorter needle-shaped crystals of taxifolin were obtained (Figure 1e).

The effect of the solution flow rate on the fiber size was evaluated. As illustrated in Figure 1f,g, the solution flow rate does not present a significant influence on the morphology of the fibers. We obtained needle-shaped crystals of taxifolin of a length of only 5 μm to 11 μm with different flow rate.

We also considered the concentration of the taxifolin/ethanol solution from 5 to 20 mg/mL and discuss the effect of the drug concentration on the fibers distribution. As shown in Figure 1h, we found needle-like crystals of 2–4 μm length with a solution concentration of 5 mg/mL. We got rod-like shape crystals (Figure 1i) where the length was increased to 24–29 μm when the solution concentration was 20 mg/mL. The size change of processed taxifolin can be explained in terms of nucleation and growth processes [25]. At a low concentration of solutions, the saturation of solutions and the precipitation of the solute occur late during the droplet expansion process; therefore, nucleation is the prevailing mechanism and we obtain smaller fibers. Conversely, at a high concentration of solutions, we got larger fibers correspondingly.

Through the above-mentioned analysis, the optimum conditions of the SAS process are the following: The temperature is 35 °C, the pressure is 25 MPa, the concentration of the taxifolin/ethanol solution is 5 mg/mL, and the solution flow rate has little effect on the particle size and shape of taxifolin. The optimum experiment is trial no. 11 in Table 1.

### 2.2. FTIR Analysis

**Figure 2:**
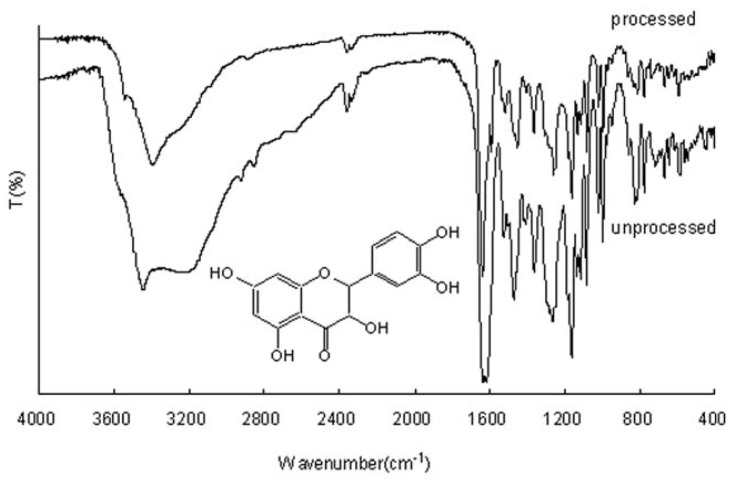
Fourier-transform infrared spectroscopy (FTIR) spectra of unprocessed and processed taxifolin (processed taxifolin is the micronized taxifolin precipitated from ethanol under optimum condition), trial no. 11 in Table 1. Insert: Chemical structure of taxifolin.

Drug structure of unprocessed and processed SAS processing was studied using FTIR spectroscopy. FTIR spectra of unprocessed and processed taxifolin are shown in Figure 2. The assignments of bands are as follows: 3420 cm^−1^ (Free O–H stretching vibration), 1620 cm^−1^ (C=O stretching in flavanones), 1360 cm^−1^ (between the O–H bending and the C–O stretching in phenolic compound), 1265 cm^−1^ (C–O–C stretching in =C–O–C– groups), 1165 cm^−1^ (a usual band in 5,7-dihydroxysubstituted flavonoids). It can be demonstrates that no significant differences are observed.

### 2.3. TG Analysis

**Figure 3:**
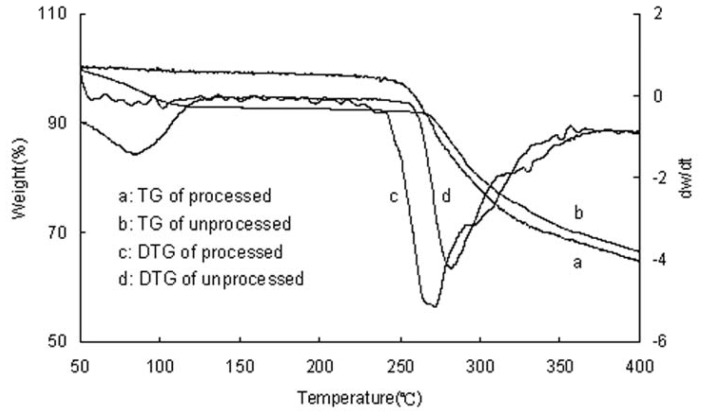
Thermal gravimetric/TG/DTG patterns of unprocessed and processed taxifolin (processed taxifolin is the micronized taxifolin precipitated from ethanol under optimum condition, trial no. 11 in Table 1).

The thermal gravimetric (TG) and differential thermal gravimetric (DTG) curves of unprocessed and processed taxifolin are shown in Figure 3. Upon heating, unprocessed taxifolin exhibited a gradual decrease in weight of about 7.86% in the region of 50–130 °C, due to the loss of some free water. On the other hand, this weight loss was not observed for the processed taxifolin in the region of 50–130 °C.

### 2.4. Powder X-ray Diffraction (XRD)

**Figure 4:**
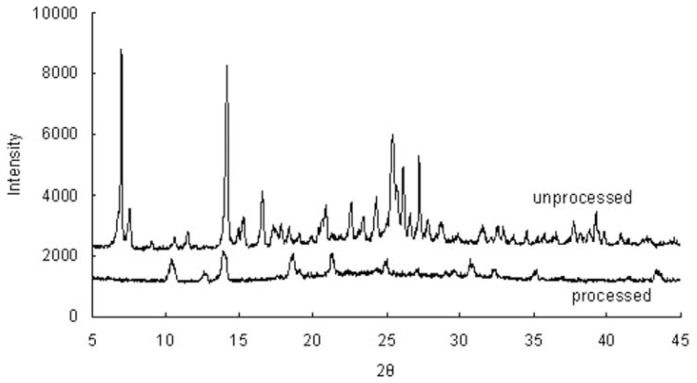
X-ray diffraction (XRD) patterns of unprocessed and processed taxifolin (processed taxifolin is the micronized taxifolin precipitated from ethanol under optimum condition, trial no. 11 in Table 1).

Figure 4 shows the X-ray diffraction (XRD) patterns of unprocessed and processed taxifolin. The characteristic high-intensity diffraction peaks of the unprocessed taxifolin revealed the existence of a relatively complete crystalline form. Intense diffraction peaks were observed at 7.06, 7.62, 14.2, 16.6 and 25.52°. However, with the processed taxifolin, the peaks at 7.06, 7.62, 14.2, 16.6 and 25.52 almost disappeared. This fact suggests that taxifolin particles existed in the amorphous state after SAS processing. It has been known that transforming the drug to an amorphous physical state leads to a high energy and high disorder state, resulting in an enhanced dissolution rate and bioavailability [26]. The presence of several distinct peaks in the XRD of processed taxifolin reveals that the drug is present in a crystalline form, but that the crystallinity is lower than that of unprocessed taxifolin. With the treatment of SAS technology, this occurred to varying degrees in the samples.

### 2.5. Solubility and Dissolution Rate

**Figure 5:**
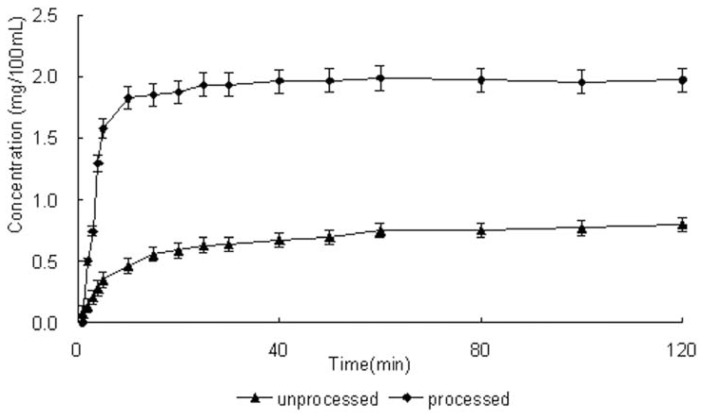
Dissolution profiles of unprocessed and processed taxifolin (processed taxifolin is the micronized taxifolin precipitated from ethanol under optimum condition, trial no. 11 in Table 1).

One purpose of preparing smaller drug particles is to increase the bioavailability of a drug by improving its dissolution rate [27]. Dissolution profiles of unprocessed taxifolin and processed taxifolin are illustrated in Figure 5. Among the samples obtained, a significant difference was observed between the unprocessed and processed taxifolin. Processed taxifolin demonstrated a higher solubility and a faster dissolution rate than unprocessed taxifolin. As can be seen, 1.6 mg/100 mL of the unprocessed taxifolin was dissolved in the first 10 minutes; while at the same time, 0.4 mg/100 mL of the processed taxifolin was dissolved. The dissolution rate of processed taxifolin is nearly four times that of unprocessed taxifolin. A higher solubility and a faster dissolution rate could be explained by the reduction of particle size resulting in an increased specific surface area. The corresponding specific surface area increased from 0.0994 m^2^/g (unprocessed taxifolin) to 2.47 m^2^/g (processed taxifolin).

### 2.6. 2,2-Diphenyl-1-picrylhydrazyl (DPPH) Radical-Scavenging Activity

**Figure 6:**
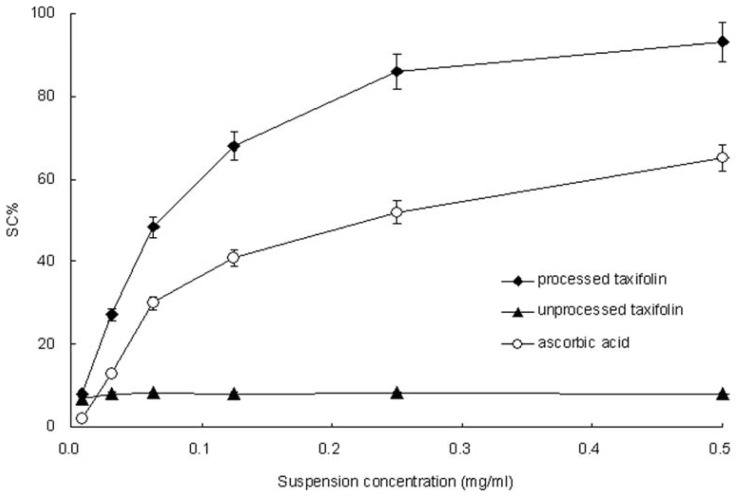
Percentage of free radical scavenging activity of unprocessed and processed taxifolin (processed taxifolin is the micronized taxifolin precipitated from ethanol under optimum condition, trial no. 11 in Table 1).

2,2-Diphenyl-1-picrylhydrazyl (DPPH) radical-scavenging activity has been extensively used for screening antioxidants. The reason for this is that the DPPH radical is not biologically relevant [28,29], and it is a useful reagent for studying the free radical-scavenging activities of compounds. DPPH free radical-scavenging activities of processed and unprocessed taxifolin are shown in Figure 6. For each sample, six concentrations were tested. Both processed and unprocessed samples showed increased free radical-scavenging activity with the increased concentration. All the samples exhibited radical-scavenging activity in the order of processed taxifolin > ascorbic acid > unprocessed taxifolin. Obviously, the results reveal that processed taxifolin exhibits considerably higher DPPH free radical-scavenging activities than unprocessed taxifolin.

## 3. Experimental Section

### 3.1. Materials

High purity CO_2_ (99.99% pure) was purchased from Liming Gas Company of Harbin (Heilongjiang, China). Absolute ethanol (analytical grade) was purchased from Sinopharm Chemical Reagent Beijing Co., Ltd (Beijing, China). Taxifolin (Purity ≥ 95%) was obtained from Ametis Company of Blagoveschensk (Amur, Russia). 2,2-diphenyl-1-picrylhydrazyl (DPPH), was purchased from Sigma Aldrich (St. Louis, MO, USA). All other solvents and chemicals used in this study were of analytical grade from Beijing Chemical Reagents Co. (Beijing, China). Deionized water was purified by a Milli-Q water purification system (Millipore, Billerica, MA, USA).

### 3.2. Preparation of Micronized Taxifolin Powder by SAS

The schematic diagram of the SAS apparatus is from Yang [30]. Briefly, SC-CO_2_ was pumped into the precipitation chamber until the desired pressure was reached. CO_2_ steady flow of 8.5 kg/h was established. Then, pure solvent was sent through the nozzle to the chamber with the aim of obtaining steady state composition conditions during the solute precipitation. At the same time, the flow of the pure ethanol was stopped and the taxifolin ethanol liquid solution was delivered into the high-pressure vessel through the stainless steel nozzle. The experiment finished when the liquid solution to the chamber was stopped. However, the supercritical CO_2_ continued flowing to wash the residual content of liquid solvent solubilized into the supercritical anti-solvent for 30 min at least. Finally, the supercritical CO_2_ flow was stopped and the precipitator chamber was gradually depressurized to atmospheric pressure, and the collected drugs were removed from the chamber for analysis. A single factor experiment was selected for optimization of operating conditions of taxifolin by SAS process. There are many factors, which affect mean particle size of taxifolin. Some of these are precipitation temperature, precipitation pressure, taxifolin solution concentration, taxifolin solution flow rate, type of solvent, composition of solvent, solvent to solid ratio, pore diameter of nozzle and CO_2_ flow rate. This study evaluated some of these important factors. We used the same solvent ethanol, pore diameter of nozzle 150 μm and CO_2_ steady flow of 8.5 kg/h for all SAS. In this study, four variables evaluated for their effects on particle characteristics and are shown in Table 1. These variables were (1) temperature, (2) pressure, (3) drug concentration and (4) drug solution flow rate.

### 3.3. Scanning Electron Microscopy (SEM)

The morphology of the unprocessed and processed taxifolin particles was performed using a SEM (Quanta 200, FEI, Hillsboro, OR, USA). Particles of representative samples were coated with gold–palladium at room temperature before examination.

### 3.4. Fourier Transforms Infrared Spectroscopy (FTIR)

The FTIR spectrum was obtained by MAGNA-IR560 E.S.P (Nicolet, Madison, WI, USA). The unprocessed and processed taxifolin particles were diluted with KBr mixing powder at 1%, pressed to obtain self-supporting disks and recorded in the wave number range of 400–4000 cm^−1^ at a resolution of 5 cm^−1^.

### 3.5. Thermal Gravimetric Analysis (TG)

Thermal gravimetric analysis was performed using a Thermo gravimetrical Analyzer (TGS-2, PerkinElmer, Norwalk, CT, USA). The experiment was performed with a heating rate of 5 °C/min using a nitrogen flow of 50 mL/min. Samples were weighed (approximately 5 mg) in open aluminum pans and the percentage of weight loss of the samples was monitored from 50 °C to 400 °C.

### 3.6. X-ray Powder Diffraction (XRD)

X-ray diffraction patterns were collected in transmission using an X-ray diffractometer with a rotating anode (Philips, Xpert-Pro, Almelo, The Netherlands) with Cu Kα_1_ radiation generated at 30 mA and 40 kV. Powders of the unprocessed and processed taxifolin were filled to the same depth inside the sample holder by leveling with spatula. The range of 2*θ* diffraction angle examined was 5–45° with steps of 0.02° and a measuring time of 0.3 s per step.

### 3.7. BET Specific Surface Area Measurement

The specific surface area of samples was determined using the method of nitrogen adsorption with a surface area analyzer (Micromeritics Instrument, Norcross, GA, USA). Calculation was based on the BET equation. The equation was developed by Stephen Brunauer, Paul Emmett, and Edward Teller and includes correctional factors that make this method incredibly useful. The equation is described as:

(1) $$\frac{P}{V(P_{0}-P)}=\frac{1}{V_{m}C}+\frac{\left(C-1\right)}{V_{m}C}\frac{P}{P_{0}}$$

where *P_0_* is the saturation pressure of the gas (atmospheric pressure if the process is carried out at the boiling point of nitrogen at atmospheric pressure), *V* is the volume of gas adsorbed per unit mass of material at pressure *P*, *V_m_* is the volume of gas required to cover a unit mass of the material with a complete monolayer of gas atoms, and C is a constant.

### 3.8. Dissolution Test

Dissolution of processed and unprocessed taxifolin particles was performed according to the USP XXV type II (paddle) method (VK 7010 dissolution apparatus, VARIAN). The test was performed at 36.5 ± 0.5 °C with a rotation speed of 100 r/min. 30 mg of drug powder was put into 1000 mL dissolution medium (6.4 g Na_2_HPO_4_·12H_2_O; 0.6 g KH_2_PO_4_; 5.85 g NaCl in 1000 mL distilled water; pH 7.4). At specific intervals, 1 mL aliquot of the dissolution medium was sampled, filtered (pore size: 0.45 μm). Filtered samples were diluted with methanol, and injected to the high performance liquid chromatography (HPLC) system for assaying the concentration of drug. Each experiment was carried out in triplicate. HPLC analyses were performed at 25 °C using the Waters HPLC system consisting of a pump (Model 1525), an auto-sampler (Model 717 plus), UV detector (Waters 2487 Dual λ absorbance detector). Separation was carried out on a C_18_ column (Diamonsil, 5 μm, 4.6 mm × 250 mm, Dikma Technologies Inc.). The mobile phase consisted of acetonitrile/water/acetic acid (82:18:0.1, v/v) and the flow rate was 1.0 mL/min. The injection volume was 10 μL and the detection wavelength was 292 nm.

### 3.9. DPPH Assay

To determine the free radical scavenging activity of each sample, the DPPH· free radical scavenging assay reported by Lu [31] was used with minor modification. The amount of 0.8, 3.125, 6.25, 12.5, 25 and 50 mg processed or unprocessed taxifolin was added to 100 mL deionized water and ultrasonically treated for 10 min to get the processed or unprocessed taxifolin suspension, respectively. The suspensions were taken through the 0.45 μm membrane. Then 0.1 mL of each sample at different suspension concentrations (0.008–0.5 mg/mL) was added to 3.9 mL of ethanol DPPH solution (25 mg/L). The absorbance was measured at 517 nm after reaction for 30 min at 20 °C in the dark. Measurements were performed at least in triplicate. The DPPH scavenging capacity (SC) of the tested samples was measured as a decrease in the absorbance and was calculated using the following equation:

(2) $$SC\%=\frac{A_{2}-A_{1}}{A_{2}}\times 100\%$$

where *A_2_* is the absorbance of the control, and *A_1_* is the absorbance of the sample.

### 3.10. Statistical Analysis

The data was subjected to analysis of variance and the significance of the difference between means was determined by Duncan’s multiple range test (*p* < 0.05) using SAS (Version 8.1, 2000; SAS Inst., Cary, NC, USA). Values expressed are means ± standard deviation.

## 4. Conclusions

Micronized taxifolin powder was successfully precipitated from ethanol by SAS. The effects of a temperature from 35 to 65 °C, a pressure range from 10 to 25 MPa, a solution flow rate from 3 to 6 mL/min and a concentration of drug solution from 5 to 20 mg/mL were considered. The precipitation temperature, pressure and concentration of taxifolin solution had a significant effect. However, the solution flow rate had a negligible effect. The length of processed taxifolin fibers was in the range of 2–300 μm with different conditions, and the width of processed taxifolin fibers was not changed obviously. A series of methods, such as SEM, FTIR, TG and BET, was used to give further analysis through observing the characterization of processed taxifolin. The dissolution rate and antioxidant activity of precipitated taxifolin were higher than those of unprocessed taxifolin.

## Figures and Tables

**Table 1:** List of experiments performed on taxifolin under different operating conditions.

Run	Pressure MPa	Temperature °C	Concentration mg/mL	Flow rate mL/min	Length μm	Width μm	Morphology
1	20	35	10	4	5–30	1.2–3.6	Needle-like
2	20	45	10	4	20–100	2.0–3.4	Needle-like
3	20	55	10	4	30–150	20.-3.6	Long needles
4	20	65	10	4	40–200	1.4–4.4	Long needles
5	10	35	10	4	10–300	2.5–3.2	Long needles
6	15	35	10	4	10–100	1.6–2.7	Needle-like
7	25	35	10	4	2–10	0.7–1.2	Needle-like
8	25	35	10	3	5–9	1.2–1.6	Needle-like
9	25	35	10	5	6–10	1.2–1.5	Needle-like
10	25	35	10	6	9–11	1.1–1.3	Needle-like
11	25	35	5	3	2–11	1.1–1.2	Needle-like
12	25	35	15	3	13–18	1.4–2.6	Rod-like shape
13	25	35	20	3	24–29	2.4–4.6	Long rod-like shape
